# *Moringa stenopetala* leaf as an alternative protein source to soybean meal in laying hens’ nutrition: Assessing its effect on egg production and egg quality

**DOI:** 10.1016/j.psj.2026.106613

**Published:** 2026-02-10

**Authors:** Kibru Beriso, Vera Sommerfeld, Markus Rodehutscord, Aberra Melesse

**Affiliations:** a School of Animal and Range Sciences, College of Agriculture, Hawassa University, Ethiopia; b Institute of Animal Science, University of Hohenheim, Germany

**Keywords:** Chicken, Egg production, Egg quality, Daily feed intake, *Moringa stenopetala*

## Abstract

The aim of this study was to investigate the effects of substituting soybean meal (**SBM**) with *Moringa stenopetala* leaf (**MSL**) on egg production and egg quality in hens from 21 to 41 wk of age. Diets were formulated to contain MSL at a rate of 0 (MSL0), 3 (MSL3), 8 (MSL8), and 13% (MSL13) by replacing SBM. Each of the four treatments was assigned to four replicate pens with ten hens each. Hen-housed egg production (**HHEP**), daily egg mass (**DEM**), and feed conversion ratio (**FCR**, g feed/g DEM) were computed. Egg quality traits were determined at 25, 29, 33, 37, and 41 wk. Replacement of SBM with MSL increased (*P* < 0.05) HHEP, DEM, and ADFI. The HHEP for MSL8 and MSL13 was higher (*P* < 0.05) than that of MSL0 and MSL3. The highest DEM was observed in MSL8 and MSL13 and differed (*P* < 0.05) from MSL3 and MSL0. A linear effect was found for HHEP, DEM, and ADFI (*P* < 0.001). A significant interaction of age by dietary level was detected for shell thickness and internal egg qualities. At 25, 33, and 41 wk, shell thicknesses of MSL3, MSL8, and MSL13 groups was higher (*P* < 0.05) than that of the control. Egg length, breadth and shell weight increased from wk 25 to 41 (*P* < 0.05). At 41 wk, albumen height and Haugh unit (**HU**) were higher (*P* < 0.05) in MSL3, MSL8, and MSL13 groups compared to MSL0. At 33 and 37 wk, yolk height was higher (*P* < 0.01) for MSL13 than others. Yolk index was greater (*P* < 0.01) for MSL13 than other groups at 33, 37, and 41 wks. The deepest yolk colour was observed in MSL13 group, followed by MSL8 and MSL3 across ages (*P* < 0.001). From wk 25 to 41, albumen height, HU, yolk height, and index decreased (*P* < 0.05). Yolk height, index, and colour linearly increased across substitution levels (*P* < 0.001). In conclusion, replacing SBM with MSL positively influenced egg production and quality, and MSL can be included in layer hen diets.

## Introduction

Poultry meat and eggs are sources of high-quality protein, providing essential amino acids that support the growth, development, and overall health of humans across all age groups. As a result, there has been an increasing demand for poultry products in many developing countries. However, the livestock sector in many developing countries cannot meet this growing demand mainly due to the limited availability of animal products and the high prize of feed resources. Soybean meal **(SBM**) is an ideal candidate for fulfilling protein requirements in poultry due to its amino acid profile (Liu et al., 2024). However, the continued rise in the price of SBM in the global market has further compelled producers to seek low-cost, readily available plant-based alternative protein sources for poultry diets with a lower carbon footprint and reduced land use. Among locally available unconventional protein sources, *Moringa stenopetala* leaves (**MSL**) could be used as an alternative feed resource in non-ruminant animals. This tree is endemic in southern Ethiopia and northern Kenya and grows all year round in the lowland agroclimatic area between 500 and 1700 meters above sea level (Melesse et al., 2012). Demisse et al. (2024) reported that approximately 96% of households in the Moringa-growing regions of southern Ethiopia supplied the edible parts of the tree, mainly leaves, to their livestock.

Global interest has emerged in implementing low crude protein **(CP**) diets for poultry. The necessity of their development stems from low-cost formulation, maintaining egg quality, reducing environmental impacts, and health and welfare concerns in egg production (He et al., 2021; Iqbal et al., 2025). Laying hens fed a low CP diet supplemented with free amino acids excreted less nitrogen in the manure while increasing egg productivity (Liu et al., 2024). It has been demonstrated that the CP content of a diet can be reduced provided the individual amino acid requirements of the animal are met (Adhikari et al., 2025). Many researchers have reported that the MSL are rich in CP (28.2–30%) and contain considerable amounts of macro- and micronutrients (Melesse et al., 2009, 2012; Mikore and Mulugeta, 2017; Ntshambiwa et al., 2023; Tamiru et al., 2020). Although the CP content of MSL is lower than that of SBM, it contains a well-balanced essential amino acid profile (Melesse et al., 2009, 2012). As a result, MSL has become a potential candidate as an alternative feed ingredient for livestock in general and for poultry in particular (Demisse et al., 2024).

Animal-based experiments using leaves and pods of *Moringa stenopetala* have been reported for both poultry and ruminant nutrition (Melesse et al., 2011, 2013, 2015). In poultry nutrition, most studies have been conducted on meat-type dual-purpose birds and commercial broilers. For instance, Melesse et al. (2011) reported significantly higher protein intake, average weight gain, and feed and protein efficiency in Rhode Island Red dual-purpose chickens fed graded levels of MSL compared to those fed the control diet. In another study, the performance of dual-purpose Koekoek chickens supplemented with 3, 8, 11, and 14% of MSL had higher (*P* < 0.01) weight gain, dressing percentage, and dressed carcass than those fed the control diets (Melesse et al., 2013). Apart from using MSL for meat production, only a few authors have reported on the utilization of MSL in laying hens’ diet. For instance, supplements of 1, 1.5, and 2% sundried MSL in layer diets did not affect egg production, feed conversion ratio, and egg quality traits except the yolk color, which was enhanced considerably (Tamiru et al., 2020). Sufe et al. (2023) reported that supplementation of varying levels of MSL (0, 20, and 40%) did not affect FCR, egg production, and quality traits of commercial layer chickens. Except for the two above, most studies in the literature report on the use of the related *Moringa oleifera* leaf (**MOL**) meal in egg production and for egg quality traits.

To the authors’ knowledge, there is no information in the literature on the use of MSL as an alternative protein source to soybean meal for laying hens, including its effects on egg production and quality traits. Given its potential as alternative feed in poultry nutrition, this study was conducted based on the hypothesis that the substitution of SBM with MSL may not negatively affect feed utilization, egg production, and egg quality traits. The objective of this study was thus to evaluate the potential of substituting SBM with graded levels of MSL on feed intake, feed efficiency, egg production, and external and internal egg qualities of laying hens.

## Materials and methods

### Ethical statement

The research proposal of the current work has been evaluated and approved by the Graduate Council of the School of Animal and Range Sciences of Hawassa University. An approval from the Institutional Review Board/Research Ethics Committee of the College of Agriculture, Hawassa University with ref. no. IRB CA/36/2018 was obtained and strictly adhered to. No debeaking was practiced and none of the animals were sacrificed during the experiment.

### Preparation of the feed materials

Fresh leaves of *Moringa stenopetala* were collected from Moringa trees regardless of the age of the trees during the dry season. The leaves were collected in Arba Minch, located in southern Ethiopia at GPS coordinates 6° 2′ N and 37° 33′ E, with an elevation of 1202 meters above sea level. The leaves were separated from the branches by hand. The leaves were air-dried in an area protected from direct sunlight to prevent loss of vitamins and volatile nutrients while retaining their green pigment. The drying process was completed in two periods: leaves were first air-dried at the collection site for one wk in the shade and transported to the experimental site, where they were dried for one additional wk. The average ambient temperature and humidity during drying period was 24 °C and 65%, respectively. The dried leaves were then ground by using a poultry feed miller (type AWILA, Germany) with 8 mm sieve size to produce the MSL meal. The ground meal was packed and stored in a dry place until mixed with the other feed ingredients and used for the experiment.

### Acquisition and management of experimental animals

Initially, 170 8-wk-old Lohmann-Tradition pullets were purchased from Alema PLC, a local agent specializing in producing and selling exotic chicken breeds. The pullets were transported to the research site and reared in a deep litter housing system of the poultry farm of Hawassa University. During the growing period, the pullets were provided with commercial pullets’ feed until at the age of 19 wk.

### Experimental design and management of birds

The primary ration was a white maize-soybean meal-based diet (control diet, **MSL0**) and experimental diets were formulated to contain MSL at a rate of 3 (**MSL3**), 8 (**MSL8**), and 13% (**MSL13**) by partially replacing the SBM (Table 1). Prior to feed formulation, the feed ingredients used were analyzed for ash, CP, crude fiber (**CF)**, ether extract (**EE**), calcium (**Ca**), and phosphorus (**P**). The analyzed chemical composition of the MSL and the experimental diets is shown in Table 2. At the age of 20 wk, hens were weighed individually (considered as initial body weight) and transferred to the experimental house containing pens with equal dimensions.

**Table 1:** Ingredient composition of experimental diets (%).

Feed ingredients	MSL0	MSL3	MSL8	MSL13
White-maize grain	54.5	53.5	51.5	49.5
Wheat bran	13.5	14.5	16.0	16.75
Soybean meal (45% CP)	20	17	12	7
Meat and bone meal	3.0	3.3	4.3	6.0
*Moringa stenopetala* leaf	0.0	3.0	8.0	13.0
Limestone	7.5	7.25	6.75	6.25
Layer hen premix*	0.50	0.50	0.50	0.50
L-Lysine-HCl	0.50	0.50	0.50	0.50
Salt	0.50	0.50	0.50	0.50
Total	100	100	100	100

MSL = *Moringa stenopetala* leaf.

⁎ Layer hen premix added to the diet included (per kg of diet): **Vitamins**: 10,000 IU vitamin A, 2000 IU vitamin D3, 5 mg vitamin E, 2.0 mg vitamin K3, 1.5 mg vitamin B1, 5.0 mg vitamin B2, 3 mg vitamin B6, 20 mcg vitamin B12, 30 mg Niacin, 1.0 mg Folic acid, 200 mg Choline chloride, 9.0 mg Calcium-d-pantothenate. **Compounds of trace minerals**: Iron 45 mg, Copper 5 mg, Manganese 60 mg, Zinc 70 mg, Iodine 1.0 mg, Selenium 0.4 mg.

**Table 2:** Analyzed compositions of treatment diets and *Moringa stenopetala* leaf meal (%, DM basis).

Treatment diets and MSL	DM	Ash	CF	CP	EE	NFE	ME (kcal/kg)
MSL0	89.1	3.93	5.8	17.7	6.0	66.6	2771
MSL3	89.3	4.10	6.3	17.5	6.3	65.8	2770
MSL8	89.1	4.53	6.2	17.3	6.2	65.8	2771
MSL13	89.5	5.13	6.6	17.4	6.0	64.9	2777
MSL	89.3	10.1	7.6	29.0	8.8	44.5	3187

MSL = *Moringa stenopetala* leaf; CP = crude protein; EE = ether extract; CF = crude fiber; NFE = nitrogen free extract; ME was calculated from the ME content of each feed ingredient used for ration formulation.

Soybean meal was substituted with *Moringa stenopetala* leaf meal (MSL) with the following dietary levels: 0, 3, 8, and 13g/100 g (MSL0, MSL3, MSL8, and MSL13, respectively).

Each of the four treatment diets was randomly assigned to four replicate pens (experimental units), with ten hens housed in each pen in a completely randomized design. Birds had unrestricted access to mash feed and water during the experimental period. All birds were managed under the same environmental and husbandry conditions during the experiment. The experiment was conducted in an open-sided naturally ventilated deep litter poultry housing system. The concrete floor was covered with wood shavings at a depth of about 5 cm and replaced with new shavings when necessary.

### Data collection procedures

***Performance traits***. Data on egg number and egg weight were collected daily from the 21st to the 41st wk of hen’s age. Feed intake of each replicate (pen) was determined by subtracting the weight of the feed refused from the total feed offered. It was recorded daily throughout the experimental period. This practice enabled precise tracking of feed intake patterns and consistency among treatment groups. Hen housed egg production (**HHEP**), daily egg mass (**DEM**), and feed conversion ratio (**FCR**, g feed/g DEM) were computed. The ADFI per hen was computed by dividing the total feed intake of each pen with number of hens per pen multiplied by number of experimental days. Hen-housed egg production (%) was computed by dividing the total egg number per pen with the number of hens per pen that were initially housed times number of days in lay multiplied by hundred. Egg mass was computed by multiplying HHEP with the average weight of eggs divided by 100. At the end of the experiment (41 wks of age), the body weight of individual hens was recorded (considered as final body weight).

***Egg quality traits****.* All eggs were counted and weighed daily, and the average egg weight per pen was calculated. Egg quality traits were evaluated at 25, 29, 33, 37, and 41 wk of age. A total of 1620 eggs were utilized for all age points. At each age point, 81 eggs from each treatment were used (81 eggs × 4 diets × 5 age points). Individual eggs were weighed on a digital balance with 0.01 g accuracy. The length and breadth of the eggs were measured with an electronic sliding caliper with 0.01 mm precision. Then, each egg was broken out on a flat glass surface. The heights of the albumen and yolk were measured using a tripod micrometer gauge while the yolk width was determined by an electronic sliding caliper. Yolk color was determined according to the Roche yolk color fan, ranging from pale yellow (1) to deep orange (16). The inside of the shell was thoroughly cleaned with tissue paper and dried for 48 h at room temperature. The dried shell was weighed on a digital balance with an accuracy of 0.01 g. Shell thickness was then determined by averaging measurements from the center, broad, and narrow ends, inclusive of shell membrane, using a digital caliper. The outer shell membrane was retained to ensure that any potential effects of MSL on it were not ignored.

Shape index was calculated as the ratio of egg breadth (**B**) to length (**L**) times 100. The egg surface area (**ESA**) was calculated as 0.933 * B (*B* + 2.343 * L) according to Narushin et al. (2021a). The volume of eggs was computed with the formula 0.5202 * L * B^2^ - 0.4065, as proposed by Narushin et al. (2021a). The eggshell ratio (shell percentage) was determined by dividing the shell weight by the egg weight times 100. The yolk index was computed by dividing the yolk height by its width times 100. The HU score was calculated according to Haugh (1937). All egg quality measurements were taken by the same person throughout the experimental period.

***Nutrient analysis****.* For chemical analyses, all samples were ground through a sieve with a pore size of 0.5 mm (Siebtechnik GmbH, Mühlheim-Ruhr, Germany, and Retsch GmbH, Haan, Germany). Analyses of proximate nutrients and minerals in feed ingredients, diets, and eggshell samples were performed as outlined by Verband Deutscher Landwirtschaftlicher Untersuchungs- und Forschungsanstalten (VDLUFA, 2007). The samples were analyzed for DM (method 3.1), ash (method 8.1), CP (method 4.1.1), CF (method 6.1.1), and EE (method 5.1.1). The nitrogen-free extract (NFE) was calculated using the following formula: % NFE = 100 – (% CP + % EE + % CF + % Ash). Calcium, P, magnesium (Mg), potassium (K), sulfur (S), sodium (Na), iron (Fe), copper (Cu), zinc (Zn) and manganese (Mn) were determined according to the methods 10 and 11 of VDLUFA (2007) using an Inductively Coupled Plasma spectrometer (ICP-OES). All analyses were run in duplicate.

### Statistical analysis

All statistical analyses were conducted using SAS (SAS, 2016, ver. 9.4). All data were first checked for normality using univariate analysis. Each treatment was replicated four times, and the experimental unit was a pen (replication) with ten hens. Initial and final body weight of individual hens was analyzed using PROC MIXED by fitting substituting levels as fixed effect and pen as random effect with individual hens being nested within pens. Average daily feed intake, HHEP and DEM data were analyzed using PROC MIXED by fitting substitution levels, as fixed effect and replicate pen within levels was included as a random effect. Week was treated as a repeated measure with pen as the subject. To visualize age dependent responses of selected performance traits, data were analyzed using the SGPLOT procedure. Additionally, a linear regression analysis was conducted to evaluate the relationship between feed intake and selected egg production traits.

Egg quality data were analyzed with PROC MIXED by fitting MSL substitution levels, age points (wk), and their interaction (levels * wk) as fixed effects. Replicate pen within levels was included as a random effect and wk was considered as a repeated measure with pen as the subject. The covariance structure providing the best fit was selected based on the lowest Akaike information criterion. The polynomial orthogonal contrast analysis was used to evaluate the linear and quadratic effects of substitution levels for egg production and egg quality traits. For all post-hoc analyses, least square means were compared pairwise with adjusted Tukey-Kramer test. Statistical significance for all test was set at *P* < 0.050, with trends at *P* < 0.100. All results were presented as least square means with SEM.

## Results

### Nutrient compositions of MSL and experimental diets

As shown in Table 2, MSL contained a high concentration of CP and ash. The analyzed CP and calculated ME concentrations of the diets were comparable among substitution levels. As presented in Table 3, the concentration of all macro minerals remained similar across substitution levels except S, which showed an increase across substitution levels. The concentrations of the micro minerals varied across substitution levels. The concentration of Fe, Zn and Mn slightly reduced across substitution levels. The analyzed macro and micro mineral compositions of eggshell at different substitution levels is presented in Table 4.

**Table 3:** Analyzed macro and micro mineral compositions of experimental diets.

Minerals	MSL0	MSL3	MSL8	MSL13
*Macro minerals* (g/kg DM)				
Calcium	32.8	32.4	32.5	33.1
Phosphorus	7.5	7.5	7.5	8.2
Potassium	10.2	10.7	10.2	10.2
Magnesium	3.2	3.3	3.2	3.4
Sulphur	2.3	2.6	3.3	3.8
Sodium	3.4	3.2	3.5	3.5
*Micro minerals* (mg/kg DM)				
Iron	1231	1129	1017	864
Zinc	207	163	188	164
Manganese	183	119	165	150
Copper	16.1	14.3	16.4	16.0

MSL = *Moringa stenopetala* leaf.

Soybean meal was substituted with MSL with the following dietary levels: 0, 3, 8, and 13g/100 g (MSL0, MSL3, MSL8, and MSL13, respectively).

**Table 4:** Analyzed macro and micro mineral compositions of eggshells from hens fed different substitution levels of soybean meal with *Moringa stenopetala* leaf meal.

Minerals	MSL0	MSL3	MSL8	MSL13
*Macro minerals* (g/kg DM)				
Calcium	348	352	353	352
Phosphorus	1.03	1.14	1.05	1.05
Potassium	0.59	0.57	0.54	0.53
Magnesium	3.9	3.9	3.6	3.7
Sulphur	1.3	1.3	1.1	1.2
Sodium	1.1	1.2	1.1	1.1
*Micro minerals* (mg/kg DM)				
Iron	34.0	41.0	34.5	35.1
Zinc	< 4	< 4	< 4	< 4
Manganese	< 0.2	< 0.2	< 0.2	< 0.2
Copper	< 0.5	< 0.5	< 0.5	< 0.5

MSL = *Moringa stenopetala* leaf; LOQ for Zn = 4, for Mn = 0.2, for Cu = 0.5.

Soybean meal was substituted with MSL with the following dietary levels: 0, 3, 8, and 13g/100 g (MSL0, MSL3, MSL8, and MSL13, respectively).

### Feed consumption and egg production

As shown in Table 5, the initial body weight of hens was similar across treatments. Hens fed the MSL13 diet had a higher final body weight (*P* < 0.05) than those fed MSL8. The final body weight showed a quadratic effect (*P* = 0.010). Except egg weight, all variables were influenced by substitution levels (*P* < 0.05). The HHEP varied among substitution levels and were higher (*P* < 0.05) in MSL8 and MSL13 groups than that of MSL3 and MSL0. The DEM of hens fed MSL8 and MSL13 diets was higher (*P* < 0.05) than that of MSL0 and MSL3. Both HHEP and DEM increased linearly with increased substitution level (*P* < 0.001).

**Table 5:** Initial and final body weight, feed intake and egg production of laying hens from 21 to 41 wk of age as affected by the substitution of soybean meal with *Moringa stenopetala* leaf meal (*n* = 4).

MSL levels	IW (g)	FW (g)	HHEP (%)	Egg weight	DEM (g/hen)	ADFI (g/hen)	FCR (g/g)
MSL0	1383	1628^ab^	56.3^b^	55.2	31.2^b^	97.6^d^	3.27^b^
MSL3	1373	1585^ab^	58.0^b^	55.2	32.1^b^	102^cd^	3.29^b^
MSL8	1383	1569^b^	64.7^a^	55.5	36.0^a^	108^bc^	3.09^c^
MSL13	1394	1683^a^	64.8^a^	54.8	35.6^a^	117^ab^	3.42^ab^
SEM	21.4	29.7	1.65	0.22	0.89	2.8	0.063
*P* values							
MSL levels	0.916	0.026	<0.001	0.904	<0.001	<0.001	0.024
Linear effect	-	0.269	<0.001	0.525	<0.001	<0.001	0.579
Quadratic effect	-	0.010	0.560	0.303	0.430	0.056	0.073

a-c Means within a row with different superscripts are significantly different (*P* < 0.05) MSL = *Moringa stenopetala* leaf; IW = initial hens’ body weight; FW = final hens’ body weight; HHEP = hen-housed egg production; DEM = daily egg mass; FCR = feed conversion ratio (g feed/g egg mass); Soybean meal was substituted with MSL with the following dietary levels: 0, 3, 8, and 13 g/100 g (MSL0, MSL3, MSL8, and MSL13, respectively).

**Fig. 1:**
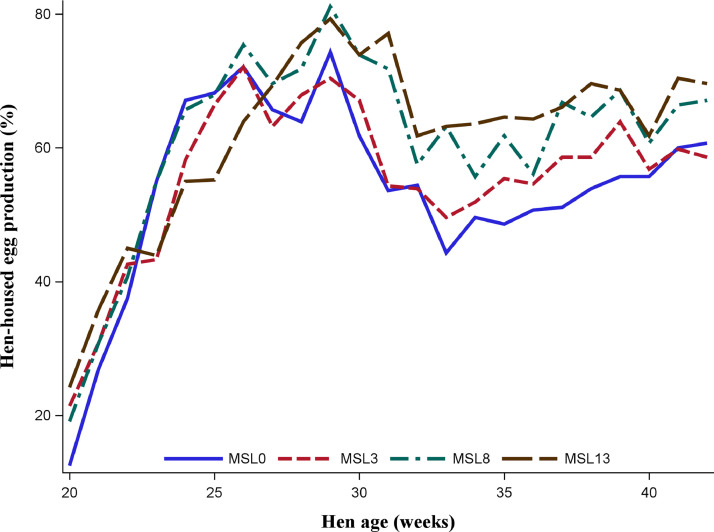
Pattern of hen-housed egg production in response to partial substitution of soybean meal with *Moringa stenopetala* leaf over the production period (21-41 wk); MSL = *Moringa stenopetala* leaf; MSL0 = control diet; MSL3 = soybean meal substituted with 3% MSL; MSL8 = soybean meal substituted with 8% MSL; MSL13 = soybean meal substituted with 13% MS.

**Fig. 2:**
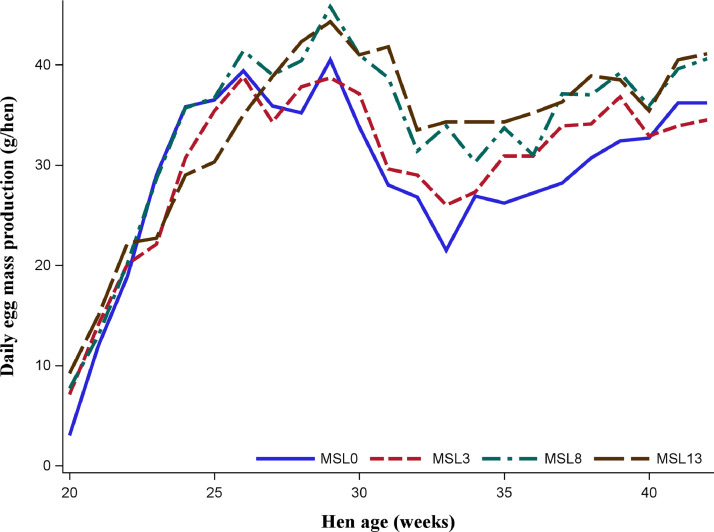
Variation in daily egg mass production of hens fed graded levels *Moringa stenopetala* leaf as a substitute for soybean meal with hens’ age; MSL = *Moringa stenopetala* leaf; MSL0 = control diet; MSL3 = soybean meal substituted with 3% MSL; MSL8 = soybean meal substituted with 8% MSL; MSL13 = soybean meal substituted with 13% MS.

**Fig. 3:**
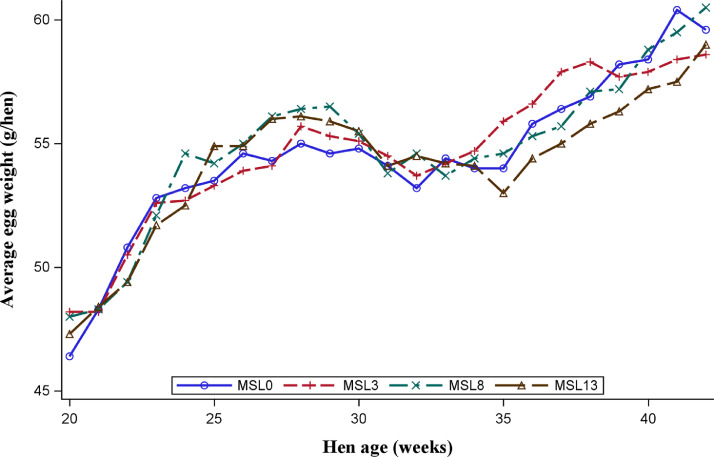
Average egg weight of hens fed different levels of *Moringa stenopetala* leaf as a partial replacement for soybean meal during the production period; MSL *= Moringa stenopetala* leaf*;* MSL0 = control diet; MSL3 = soybean meal substituted with 3% MSL; MSL8 = soybean meal substituted with 8% MSL; MSL13 = soybean meal substituted with 13% MSL.

**Fig. 4:**
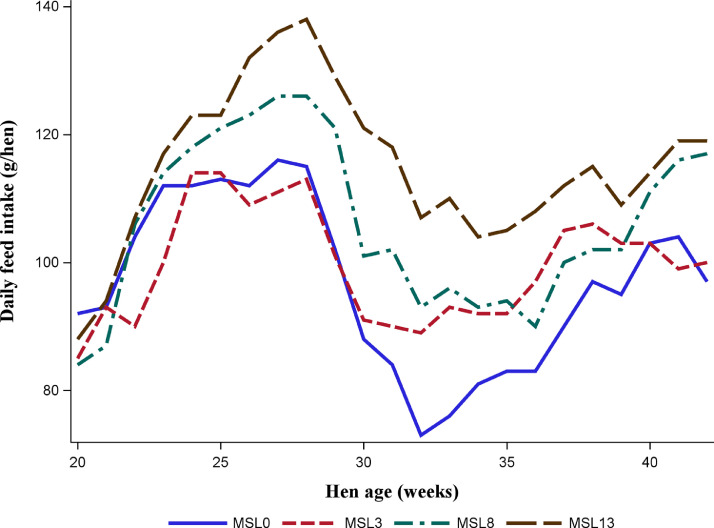
The effect of graded levels of *Moringa stenopetala* leaf as a substitute for soybean meal on average daily feed intake in respect to advancing age; MSL = *Moringa stenopetala* leaf; MSL0 = control diet; MSL3 = soybean meal substituted with 3% MSL; MSL8 = soybean meal substituted with 8% MSL; MSL13 = soybean meal substituted with 13% MSL.

**Fig. 5:**
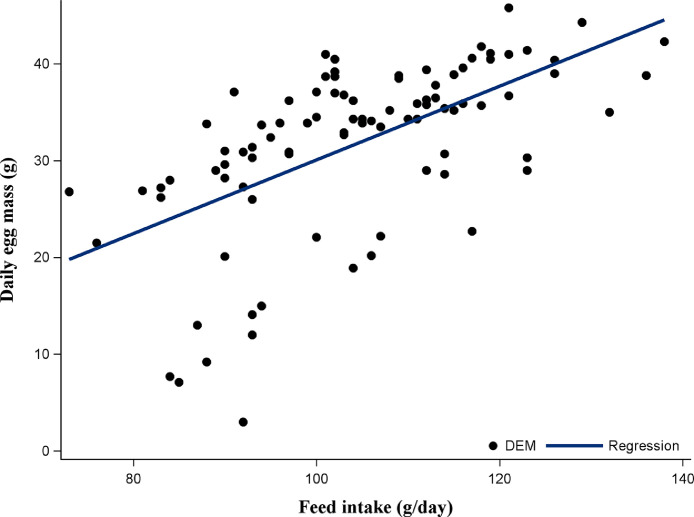
Relationship between average daily feed intake and daily egg mass production of hens fed graded levels of *Moringa stenopetala* leaf as a substitute for soybean meal (*Y* = 0.708 x - 23.7, R^2^ = 0.35); Y = daily egg mass (DEM); x = daily feed intake.

**Fig. 6:**
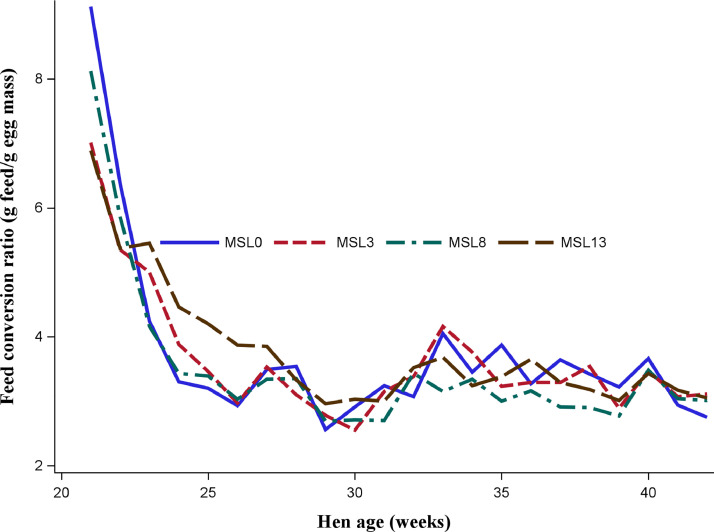
Pattern of feed conversion ratio of hens fed different levels of *Moringa stenopetala* leaf by replacing soybean meal over the production period.

As shown in Figs. 1 and 2, the data on HHEP and DEM exhibited changes during the experimental period, with a similar pattern across substitution levels. Both performance traits reached the peak at about 29 wk of age, then dropped to 33 wk, and slightly rose again. Fig. 3 shows that the average egg weight increased consistently with the age of the hens. The ADFI was highest for MSL13 group and differed (*P* < 0.05) from that of MSL3 and MSL0. The ADFI linearly increased with increasing MSL levels (*P* < 0.001) (Table 5). It also increased with advancing age and reached its peak at about 29 wk of age and then gradually dropped to 33 wk and rose again (Fig. 4). The DEM was associated with the ADFI of the hens (Fig. 5). It showed an increasing trend with increased ADFI. The lowest FCR was obtained from hens fed the MSL8 diet and it differed (*P* < 0.05) from those fed the other diets. As shown in Fig. 6, the FCR was highest during the start of the egg-laying period and then consistently decreased until the age of 25 wk, after which it was stabilized.

### Egg quality traits

***External egg qualities*.** The effect of MSL, age, and their interaction with external egg qualities is presented in Table 6. The interaction between substitution levels and age of the hen was significant for shell thickness only. At 25, 33, and 41 wks of age, hens receiving MSL3, MSL8, and MSL13 diets exhibited comparable shell thickness, which was higher (*P* < 0.05) than that observed in the control diet group. At 29 wk, the shell thickness observed in the MSL8 and MSL13 groups was comparable; however, both were greater (*P* < 0.05) than those measured in the MSL3 and the control diet groups. Except for egg breadth and shape index, other external egg qualities were affected by substitution levels (*P* < 0.05). The highest values for egg length, egg surface area, and volume were observed in hens fed MSL8, being higher (*P* < 0.05) than those fed MSL0 and MSL3 diets. The values for dry shell weight, and shell ratio were similar for hens fed MSL3, MSL8, and MSL13 diets but were higher (*P* < 0.05) than those fed MSL0. Shell thickness and dry shell weight increased linearly with increasing substitution levels (*P* < 0.001 and *P* = 0.004, respectively). A significant quadratic effect was also observed for dry shell weight (*P* < 0.001), shell ratio (*P* = 0.002), and shell thickness (*P* < 0.001). Except for the shell ratio, the effect of age was also significant for other quality traits. Accordingly, egg length and breadth, dry shell weight, egg surface area and egg volume increased from wk 25 to 41 *(P* < 0.05).

**Table 6:** Least square means of external egg quality traits measured at different age points as affected by substitution of soybean meal with *Moringa stenopetala* leaf, hen’s age, and their interactions from 21 to 41 wk of age (*n* = 4).

Factors	MSL levels	EL (mm)	EB (mm)	SI (%)	SW (g)	STH (mm)	SR (%)	ESA	Vol
Age (wk)									
25	MSL0	53.7	42.3	78.6	5.14	0.324^b^	9.47	66.3	49.9
	MSL3	53.2	42.1	79.0	5.29	0.350^a^	9.82	65.5	49.1
	MSL8	53.7	42.1	78.3	5.25	0.348^a^	9.70	65.9	49.5
	MSL13	53.5	42.1	78.8	5.13	0.347^a^	9.46	65.8	49.3
29	MSL0	53.2	42.0	79.1	5.20	0.314^b^	9.64	65.4	49.9
	MSL3	53.4	42.2	79.0	5.35	0.328^b^	9.74	65.9	49.1
	MSL8	54.4	42.8	78.8	5.59	0.349^a^	9.73	68.0	49.5
	MSL13	53.9	42.5	78.7	5.48	0.358^a^	9.67	66.9	49.3
33	MSL0	54.2	42.0	77.5	5.12	0.347^b^	9.21	66.2	49.8
	MSL3	53.6	42.1	78.3	5.56	0.383^a^	10.00	65.6	49.2
	MSL8	54.1	42.3	78.2	5.55	0.378^a^	9.70	66.7	50.4
	MSL13	53.9	42.2	78.4	5.59	0.379^a^	9.86	66.5	50.2
37	MSL0	54.2	42.8	78.9	5.58	0.333	9.66	67.8	51.6
	MSL3	54.7	43.0	78.6	5.69	0.335	9.73	68.6	52.6
	MSL8	54.8	42.9	78.4	5.67	0.336	9.85	68.6	52.5
	MSL13	54.4	42.5	78.3	5.56	0.338	9.81	64.4	51.1
41	MSL0	55.6	43.9	79.0	5.87	0.321^b^	9.54	71.4	55.8
	MSL3	55.7	43.6	78.3	5.92	0.341^a^	9.82	70.8	55.1
	MSL8	56.1	44.0	78.5	6.07	0.348^a^	9.74	72.2	56.7
	MSL13	55.8	43.6	78.2	5.87	0.346^a^	9.68	71.0	55.3
pooled SEM		0.13	0.09	0.18	0.043	0.002	0.065	0.27	0.31
MSL levels									
MSL0		54.2^b^	42.6	78.6	5.38^b^	0.327	9.51^b^	67.4^b^	51.2^b^
MSL3		54.1^b^	42.6	78.7	5.56^a^	0.347	9.82^a^	67.3^b^	51.1^b^
MSL8		54.6^a^	42.8	78.4	5.62^a^	0.352	9.74^a^	68.3^a^	52.2^a^
MSL13		54.3^ab^	42.6	78.5	5.53^a^	0.354	9.70^a^	67.5^ab^	51.3^ab^
*P* values									
MSL levels		0.019	0.112	0.675	<0.001	<0.001	0.001	0.019	0.021
Age		<0.001	<0.001	0.026	<0.001	<0.001	0.586	<0.001	<0.001
MSL x Age		0.370	0.282	0.619	0.087	<0.001	0.301	0.269	0.283
Linear effect of levels		0.122	0.523	0.320	0.004	<0.001	0.054	0.240	0.269
Quadratic effect of levels		0.292	0.238	0.980	<0.001	<0.001	0.002	0.194	0.179

a,b Means within a row with different superscripts are significantly different (*P* < 0.05) MSL = *Moringa stenopetala* leaf; EL = egg length; EB = egg breadth; SI = shape index; SW = shell weight; STH = shell thickness; SR = shell ratio; ESA = eggshell surface; Vol = egg volume Soybean meal was substituted with MSL with the following dietary levels: 0, 3, 8, and 13 g/100 g (MSL0, MSL3, MSL8, and MSL13, respectively).

***Internal egg qualities*.** The interaction of age by substitution levels was significant for all internal quality traits (Table 7). At 37 wk of age, both albumin height and HU values, were higher for MSL3 and MSL13 groups and differed (*P* < 0.05) from those of MSL0. By 41 wk, these parameters remained higher (*P* < 0.05) in the MSL3, MSL8, and MSL13 groups compared to MSL0. Hens fed the MSL13 diet exhibited increased yolk height (*P* < 0.05) at both, 33 and 37 wks, when compared with other dietary levels. At 37 wk, hens fed MSL0, MSL3, MSL8 diets produced eggs with higher yolk width than those raised in the MSL13 diet (*P* < 0.05). The yolk index was consistently higher (*P* < 0.05) in hens fed the MSL13 diet than those fed other diets at 33, 37, and 41 wks. Both MSL3 and MSL8 exhibited higher (*P* < 0.001) yolk index than the MSL0 at 41 wk. Yolk colour also demonstrated a strong difference (*P* < 0.001) between substitution levels; the most intense pigmentation being observed in hens fed the MSL13 diet, followed by those on the MSL8, MSL3, and control diets across all evaluated time points. A strong linear effect was observed for yolk height (*P* < 0.001), yolk index (*P* < 0.001), and yolk color (*P* < 0.001). A quadratic effect was also observed for yolk width (*P* = 0.007), yolk index (*P* = 0.026), and yolk color (*P* < 0.001).

**Table 7:** Effect of substitution of soybean meal with *Moringa stenopetala* leaf, fed from 21 to 41 wk of age, hen’s age and their interactions on internal egg qualities measured at different age points (mm, unless indicated otherwise) (*n* = 4).

Factors	MSL levels	Albumen height	HU	Yolk height	Yolk width	Yolk index	Yolk colour
Age (wk)							
25	MSL0	8.60	93.9	18.3	38.1	48.3^b^	2.79^d^
	MSL3	8.52	93.5	18.2	37.5	48.5^b^	5.35^c^
	MSL8	8.43	93.0	18.5	37.1	50.1^a^	6.86^b^
	MSL13	8.15	91.6	18.4	37.3	49.2^ab^	7.75^a^
29	MSL0	9.01	95.9	18.5	36.9^b^	50.1	2.37^d^
	MSL3	9.13	96.3	18.3	37.0^a^	49.5	5.41^c^
	MSL8	9.01	95.2	18.6	37.8^a^	49.3	6.89^b^
	MSL13	8.97	95.1	18.7	37.1^b^	50.4	8.21^a^
33	MSL0	7.84	89.6	17.4^bc^	37.5^a^	46.4^ab^	1.70^d^
	MSL3	7.82	89.5	17.2^c^	38.0^ab^	45.0^c^	4.96^c^
	MSL8	7.88	89.3	17.6^b^	38.4^a^	45.8^bc^	7.28^b^
	MSL13	8.01	90.2	17.9^a^	38.0^ab^	47.2^a^	8.62^a^
37	MSL0	7.48^b^	86.9^b^	16.8^c^	39.0^ab^	43.1^c^	1.72^d^
	MSL3	8.17^a^	90.5^a^	17.0^bc^	39.5^a^	42.9^c^	5.19^c^
	MSL8	7.77^ab^	88.6^ab^	17.2^b^	38.7^b^	44.4^b^	7.30^b^
	MSL13	8.19^a^	91.1^a^	17.5^a^	38.0^c^	46,0^a^	8.47^a^
41	MSL0	7.65^b^	86.9^b^	16.7^b^	39.8	42.0^c^	1.73^d^
	MSL3	8.21^a^	90.3^a^	17.4^a^	39.8	43.7^b^	5.11^c^
	MSL8	8.23^a^	89.9^a^	17.7^a^	39.9	44.3^ab^	7.06^b^
	MSL13	8.34^a^	90.9^a^	17.6^a^	39.5	44.7^a^	9.38^a^
Pooled SEM		0.071	0.38	0.05	0.10	0.002	0.032
MSL levels							
MSL0		8.11	90.7	17.5	38.3	45.9	2.06
MSL3		8.37	92.0	17.6	38.4	45.9	5.21
MSL8		8.27	91.2	17.9	38.4	46.7	7.08
MSL13		8.33	91.8	18.0	38.0	47.5	8.48
*P* values							
MSL levels		0.027	0.020	<0.001	0.007	<0.001	<0.001
Age		<0.001	<0.001	<0.001	<0.001	<0.001	<0.001
MSL x Age		0.005	0.001	<0.001	<0.001	<0.001	<0.001
Linear effect of levels		0.058	0.089	<0.001	0.061	<0.001	<0.001
Quadratic effect of levels		0.151	0.266	0.794	0.007	0.026	<0.001

a-d Means within a row with different superscripts are significantly different (*P* < 0.05) MSL = *Moringa stenopetala* leaf; Soybean meal was substituted with MSL with the following dietary levels: 0, 3, 8, and 13g/100 g (MSL0, MSL3, MSL8, and MSL13, respectively).

## Discussion

Among the 13 Moringa species studied by Olson et al. (2016), the highest total and soluble protein concentrations were observed in *Moringa stenopetala* (29.7 and 28.6%, respectively). The CP concentration of MSL in the present study is consistent with the findings reported by Mikore and Mulugeta (2017) and Tamiru et al. (2020) (28.7 and 29.0%, respectively) for the same Moringa species. Slightly lower CP and EE values for MSL than those found in the present study were reported by Ntshambiwa et al. (2023) and Befa et al. (2020). The CF and EE concentrations of MSL reported by Tamiru et al. (2020) were higher than observed in the present study. Ntshambiwa et al. (2023) found 43.4% NFE for MSL which is consistent with the value found in the present study (44.0%). Nitrogen free extract is a calculated value and contains soluble carbohydrates, such as starch and sugar. Melesse et al. (2019) reported 44.0% of starch concentration in MSL which is almost the same as the calculated NFE (44.5%) in the current study (Table 2). Differences in nutrient concentrations of the same Moringa species might be attributed to the age of the tree, season when leaves were collected, and agroecology in which the Moringa plant has been cultivated (Desalegne et al., 2019; Melesse et al., 2012; Mikore and Mulugeta, 2017).

### Feed intake and egg production

Sufe et al. (2023) reported that using varying levels of fresh MSL in laying hen diets did not influence total egg production, DEM, and HHEP, which contrasts with the results of the present study. This might be due to the difference in CP concentrations, which was very low in the fresh MSL as compared with the one used in the present study (21.5 vs. 29.0%). The observed increase in egg production and DEM could be related to enhanced feed intake of hens which showed a linear increase with increased substitution levels (Table 5 and Fig. 4). The increase in feed consumption across substitution levels indicates the palatability of MSL when incorporated with other feed ingredients. Moreover, the improved performance of hens fed with increased levels of MSL might be related to the quality of protein provided by MSL, specifically the balance of essential amino acids it contains. The protein quality in animal feed is determined by its ability to provide essential amino acids for animal growth and function. Although the CP content of MSL is lower than that of SBM, it contains a well-balanced essential amino acid profile (Table 8). Therefore, it may serve as a promising alternative feed ingredient for livestock in general and for poultry in particular.

**Table 8:** Essential amino acid composition of soybean meal and *M. stenopetala* leaf reported from three different cited sources (g/16 g N).

Amino acids	Soybean meal	*Moringa stenopetala* leaf	
Lysine	5.78	5.68	5.51
Methionine	1.41	1.71	1.65
Cysteine	1.45	2.04	2.36
Methionine + cysteine	2.86	3.75	4.01
Leucine	7.48	8.50	7.84
Isoleucine	4.68	4.47	3.89
Phenylalanine	5.19	5.27	5.22
Valin	5.02	5.34	4.97
Threonine	3.77	4.45	4.38
Arginine	7.26	6.40	7.58
Tryptophan	N/A	1.54	N/A
Cited sources	Ravindran et al. (2014)	Melesse et al. (2009)	Melesse et al. (2012)

To the author's knowledge, there are only a few publications reporting effects of MSL on egg production and quality traits in laying hens. However, effects of MOL inclusion in laying hen diets have often been studied. Significant increases in egg number per hen, HHEP, and DEM by MSL inclusion levels obtained in the current study agree with those of Abdel-Wareth and Lohakare (2021), who reported that the inclusion of MOL in layer hen diets increased egg production. The increased egg production and egg mass might be attributed to the presence of active components in moringa leaves that possess antimicrobial and antioxidant properties as well as gastro-protective and mucus-enhancing enzyme activities (Ahmad et al., 2018). Umesh et al. (2022) reported that the use of MOL at 5, 10, and 15% in diets increased egg production, feed intake, feed efficiency, and egg weight in laying hens and the best results were obtained at 10% MOL inclusion, which agrees with the present study. Lu et al. (2016) used diets containing 5%, 10%, or 15% MOL and found no significant effects on egg weight, consistent with the present results. They reported that hens supplemented with 15% MOL had higher FCR, which is comparable to the present study, where the highest FCR was observed at 13% MSL inclusion. The same authors reported that feed intake was unaffected by MOL inclusion. However, the CP concentration of the MOL used in the reported experiment was 21.9% which was lower than that of the MSL used in the present study (29.0%). Moreover, the CF content of the MOL used in the reported experiment was higher than that in MSL (25 vs. 7.6%), which might be due to species difference, age of the tree and the season when leaves were collected (Melesse et al., 2012). The use of MOL in the diet of laying hens at levels of 1.5%, 4.5%, and 6% did not affect performance traits, but it significantly increased average egg weight (da Silva et al., 2024). It is worth noting that the CP concentration of MOL in that study was lower than that of MSL in the present study (18% vs. 29%).

### External and internal egg qualities

The enhanced yolk height and yolk index observed in the current study may be associated with the presence of selenium (**Se**) in the feed. A significant concentration of Se (3.96 mg/kg) has been documented in MSL (Kumssa et al., 2017). A recent study by Chen et al. (2025) found that organic Se (Se-C, Se-Met) and Nano-Se significantly increased the yolk index and alleviated the declines in albumen height and HU during storage. The value of the shape index for egg-producing breeds is reported to range from 73 to 80%, and for meat-type chickens from 76 to 80% (Alikhanov et al., 2021). In the present study, the shape index ranged from 78.4 to 78.7% and was not affected by substitution levels. Since most eggs are sold in their shells, the strength of the eggshell is important as a protective shield that determines the resilience of eggs during handling and transportation. The Ca concentration of MSL (2.2%, data not shown) was comparatively higher than that of SBM and this may have contributed to the observed increased eggshell quality (shell thickness, dry shell weight, and shell ratio) in MSL-fed groups. Both eggshell weight and eggshell thickness were the only external quality traits that were linearly affected by MSL substitution level (*P* = 0.004 and *P* < 0.001, respectively). Although marginally insignificant, shell ratio also showed a linear trend (*P* = 0.054). In agreement with these results, Abdel-Wareth and Lohakare (2021) reported that the eggshell thickness significantly increased as a response to diets supplemented with even low levels of MOL (0, 0.3, 0.6, and 0.9%) at 72 wk of age. In the present study, shell thickness increased by 8.3, 7.65 and 6.12% in MSL13, MSL8 and MSL3 groups as compared to the control. Siti et al. (2019) reported that shell thickness increased by 19.7% and 21.2% in 2% and 4% MOL-fed groups as compared to the control diet. They further reported that laying hens supplemented with 4% and 6% MOL responded with a higher shell thickness than those in 2% MOL-fed and control groups.

In the current study, the concentration of S in the diet increased with increasing substitution levels, suggesting MSL contains higher S than SBM. Sulphur plays a critical role in the biosynthesis of the S-containing amino acids methionine and cysteine. Both are essential amino acids involved in the synthesis of ovalbumin, a primary protein found in albumen, and act as precursors for glutathione, an antioxidant that protects cells during egg formation (Esteve‐Garcia and Llaurado, 1997). The highest S concentration was observed in *Moringa stenopetala* among 13 *Moringa* species studied (Olson et al., 2016). This all may suggest that the observed increase in albumen height and HU score might be associated with the concentrations of both methionine and cysteine in the MSL. Both traits are important indicators of egg quality and freshness (Narushin et al., 2021b). By combining egg weight and thick albumen height, the HU score offers a reliable validation of freshness. Since the HU score quickly declines over time, it is considered a sensitive measure of egg quality deterioration soon after collection. On the other hand, the yolk index quantifies the structural integrity of the egg yolk, which gradually decreases over the storage period, enabling the identification of quality differences among eggs that have deteriorated over time (Yurtseven et al., 2021).

In the current study, the highest albumen height and HU score values were obtained in hens fed MSL3, which is consistent with the findings of Voemesse et al. (2019) who reported a significant increase in the albumen height at 3% MOL supplementation. In contrast, Siti et al. (2019) found no significant differences for the albumen height and HU among groups fed with 2, 4, and 6% MOL. Similarly, Shen et al. (2021) observed no significant differences in HU score in hens fed with MOL at 0, 2.5, 5, 7.5, and 10% of feed in late laying stage. The absence of a significant effect in MOL-fed groups might be related to the age of the chickens, the quality of MOL used, and the environmental conditions in which the experiments were conducted.

The egg yolk colour is influenced by plant material type and intake of the hen, where different carotenoids of different herbs are reflected in the yolk carotenoids (Lordelo et al., 2017; Nisa et al., 2024). In commercial poultry industry of many developing countries, most poultry feed is formulated with white maize as the primary ingredient. As a result, table eggs reaching consumers lack the basic yolk color, leading to consumer rejection. Thus, searching for locally available feed material that enhances yolk color is justifiable. In the present study, the intensity of yolk color significantly increased with increased MSL substitution levels, with significant linear and quadratic effects, which is consistent with the observations of various researchers who used graded levels of MSL and MOL (da Silva et al., 2024; Shen et al., 2021; Sufe et al., 2023; Tamiru et al., 2020; Nisa et al., 2024). Siti et al. (2019) reported an increase of 29.2% and 32.6% in yolk color in hens supplemented with 2% and 4% MOL, as compared with the control group. In the present study, the yolk color increased by twofold at 3% MSL inclusion level compared to 2% MOL, which suggests that MSL may contain higher concentrations of carotenoids than MOL. Previous studies have reported that MSL is a good source of vitamin A, providing it as beta-carotene. For instance, Abuye et al. (2003) have found significant levels of beta-carotene in fresh MSL, with concentrations of 160 µg/100 g (532 IU/100 g), which is considerably higher than reported for SBM (55 IU/kg DM) (Pickworth et al., 2012). When beta-carotene is adequately present in the diet, bioactive compounds along with other phytochemicals can improve egg quality and have a positive effect on chicken health and performance (Siti et al., 2019; Ahmad et al. 2018; Nisa et al., 2024).

Egg quality traits may increase or decrease with the advancing age of the hen. Manyeula et al. (2021) reported that dry shell weight and shell ratio increased with the age of the hen, which is consistent with the present findings. They, however, noted that shell thickness remained constant with the age of birds. On the other hand, yolk height, yolk index, albumen height, and HU decreased with the advanced hens’ age, and these observations are in line with those reported by Kraus et al. (2021) and Tainika et al. (2024). Similarly, Alo et al. (2024) reported a significant decline in albumen height and HU score with increasing age of the hens.

## Conclusions

This research represents the first comprehensive investigation on the effects of incorporating MSL as an alternative protein source to SBM on feed intake, egg production and egg quality traits. Substitution of SBM with MSL was effective in enhancing the yolk color and quality of albumen and egg yolk. Although MSL contains less CP than SBM, its balanced amino acid profile confers high-quality protein benefits to small-scale poultry producers. The influence of MSL on cholesterol, triglyceride, and polyunsaturated fatty acid levels in egg yolk warrants further research.

## CRediT authorship contribution statement

**Kibru Beriso:** Writing – original draft, Investigation, Data curation. **Vera Sommerfeld:** Writing – review & editing, Supervision, Methodology. **Markus Rodehutscord:** Validation, Supervision, Resources, Methodology, Funding acquisition, Conceptualization. **Aberra Melesse:** Writing – review & editing, Validation, Supervision, Resources, Project administration, Methodology, Funding acquisition, Formal analysis, Conceptualization.

## Disclosures

The authors declare that they have no known competing financial interests or personal relationships that could have appeared to influence the work reported in this paper.
